# The gut microbiome tango in the progression of chronic kidney disease and potential therapeutic strategies

**DOI:** 10.1186/s12967-023-04455-2

**Published:** 2023-10-03

**Authors:** Zijing Tang, Shiyan Yu, Yu Pan

**Affiliations:** 1 Department of Nephrology, Shanghai Ninth People’s Hospital Affiliated with Shanghai Jiao Tong University School of Medicine, Shanghai, China; 2 Shanghai Institute of Precision Medicine, Shanghai Ninth People’s Hospital Affiliated with Shanghai Jiao Tong University School of Medicine, Shanghai, China

## Abstract

Chronic kidney disease (CKD) affects more than 10% population worldwide and becomes a huge burden to the world. Recent studies have revealed multifold interactions between CKD and gut microbiome and their pathophysiological implications. The gut microbiome disturbed by CKD results in the imbalanced composition and quantity of gut microbiota and subsequent changes in its metabolites and functions. Studies have shown that both the dysbiotic gut microbiota and its metabolites have negative impacts on the immune system and aggravate diseases in different ways. Herein, we give an overview of the currently known mechanisms of CKD progression and the alterations of the immune system. Particularly, we summarize the effects of uremic toxins on the immune system and review the roles of gut microbiota in promoting the development of different kidney diseases. Finally, we discuss the current sequencing technologies and novel therapies targeting the gut microbiome.

## Introduction

Chronic kidney disease (CKD) is one of the most important chronic diseases worldwide, affecting the well-being of many people. Currently, the main therapies for CKD are renin–angiotensin aldosterone system inhibitors with drugs for symptomatic treatment and dietary restriction. However, these interventions have achieved limited benefits. Recently, an increasing body of data has implicated that both the progression of CKD and its complications are related to the disturbed gut microbiome in patients with CKD.

**Fig. 1:**
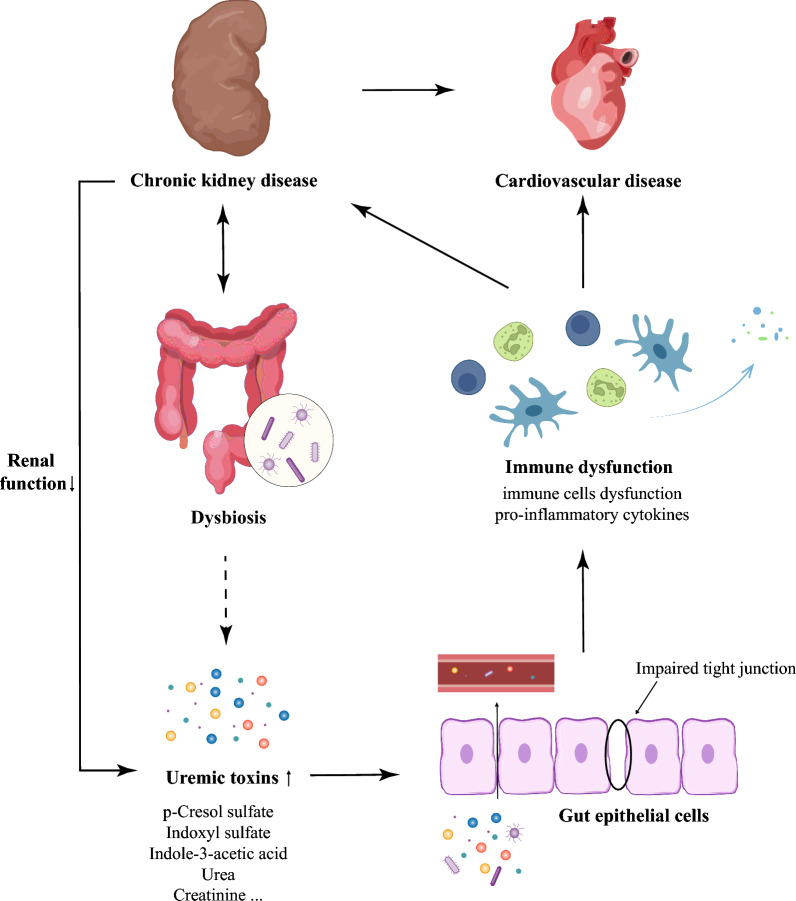
The vicious cycle between chronic kidney disease and gut dysbiosis

Gut microbiome is considered a “living organism” that coevolves with the host. Compared with the human body, it exceeds the number of cells in the human body by a factor of 10 [1]. The gut microbial genes are 100-fold greater than human genes, indicating the diversity of microbial species [2]. In healthy individuals, microbial communities as well as their metabolites perform multiple beneficial functions through bacterial-host and bacterial-bacterial interactions. The flora composition can be altered by many environmental factors such as dietary habits, drugs and disease conditions [3]. The kidney diseases, such as IgA nephropathy (IgAN), diabetic nephropathy (DN) and end-stage renal disease (ESRD) with hemodialysis (HD) or peritoneal dialysis (PD), can discompose intestinal microbial ecologies, referred to as dysbiosis [4]. Extensive efforts have been invested toward unveiling the changes. For example, Vaziri et al*.* discovered that 175 operational taxonomic units (OTUs) were different between CKD and healthy rats, and the richness of OTUs was decreased in CKD group [5]. At the family level, both *Lactobacillaceae* and *Prevotellaceae* were notably reduced, resulting in a decreased production of short chain fatty acids (SCFAs). This is attributed to the enrichment of phosphotransbutyrylase and butyrate kinase in these two taxa. Whereas the urease- and uricase-containing and indole- and p-cresol-forming intestinal microbiota increased, such as *Enterobacteriaceae*, *Pseudomonadaceae*, and *Clostridiaceae* [5, 6]. In return, gut dysbiosis is also involved in the onset and progression of CKD and complications. The potential mechanisms are associated with immune dysfunction, translocation of bacteria via impaired intestinal barriers and increased production of gut-derived uremic toxins [7, 8]. Among them, the protein-bound uremic toxins (PBUTs), such as indoxyl sulfate (IS), p-cresol sulfate (pCS) and indole-3-acetic acid (IAA), are defined as potential immune-modulatory candidates. These metabolites cause the dysfunction of immune cells and induce the production of pro-inflammatory cytokines and chemokines. The permeability of the gut barrier increases in CKD, leading to the translocation of inflammatory factors and uremic toxins into circulation. This contributes to vascular damage, glomerular sclerosis, kidney fibrosis and tubular damage, promoting inflammation and resulting in adverse outcomes and complications of CKD, such as cardiovascular disease (CVD) [9–11]. Thus, the pathological interactions between kidney disease and dysbiosis could generate vicious cycles accelerating kidney disease progression (Fig. 1).

Recent years witness the emergence of new methods, such as next-generation sequencing (NGS) and third-generation sequencing (TGS). In addition, researchers prefer to integrate multi-omics analysis to unravel the intricate interactions between the gut microbiome and CKD. These methods have improved our understanding of the gut flora ecosystem, which fuels the search for novel diagnostic methods and treatments for CKD.

In this review, we will focus on the effects of the gut microbiome on CKD progression and immune dysfunction. Additionally, advanced detection techniques and potential therapeutic avenues for CKD will also be discussed.

## Mechanisms of CKD progression

CKD is a common chronic disease with a high rate of morbidity and mortality, affecting approximately 843.6 million population worldwide [12]. Kidney Disease: Improving Global Outcomes (KDIGO) defines CKD as abnormalities of kidney structure or function, present for > 3 months, with implications for health [13]. Based on the glomerular filtration rate (GFR), CKD is classified into 5 categories. From 2017 to March 2020, the prevalence of CKD stages 1–4 was 14.7%, and the crude incidence rate of CKD stage 5, namely ESRD, will increase 11–18% from 2015 to 2030, which is a huge medical burden for the whole world [14, 15].

The mechanisms of CKD progression are enormously complex and only partly understood. Several factors have been implicated in contributing to the pathophysiological and pathological alterations observed in individuals with CKD, including but not limited to hyperglycemia, hypertension, infections, high-protein diets, and others. From a pathophysiological point of view, hyperglycemia, hypertension and immune dysfunction can directly cause cellular damage. In response to these insults, the reactive oxygen species (ROS), pro-inflammatory and pro-fibrosis factors increase in circulation, and the immune complexes deposit in the glomerulus. This leads to damage to the glomerulus and decreased GFR. The declined GFR activates the renin-angiotensin system (RAS), shown by the increasing levels of angiotensin-II to elevate the glomerular hydrostatic pressure and filtration, ultimately compromising the glomerular barrier [16]. Apart from controlling vascular pressure, angiotensin-II also induces factors that regulate inflammation and fibrosis [16]. These pathophysiological changes further result in glomerular sclerosis, tubulointerstitial fibrosis, and proliferation of mesangial cells. The pathological remodeling of kidney infrastructure is closely associated with clinical manifestations such as albuminuria and hematuria. Furthermore, uremic toxins are retained and actively participate in the processes of CKD progression stated above. The resulting disturbances in the humoral environment can impair other systems, causing CVD, bone diseases, abnormal immunity, etc. [17].

The immune dysfunction of CKD has been well reviewed elsewhere [18]. These immune abnormalities can further exert negative effects on patients with CKD. Toll-like receptor (TLR) 2 and TLR4 play a significant role in the inflammation pathway [19]. Patients with CKD exhibit the upregulation of Toll-like receptor (TLR) 2 and TLR4 on neutrophils and monocytes partly due to the elevated production of pro-inflammatory cytokines and ROS [20]. These inflammatory factors can also cause tissue impairment and are related to low GFR [21]. There is an increase in the number of peripheral polymorphonuclear leukocytes (PMNLs) in patients with CKD, which is demonstrated to be negatively correlated with GFR [22]. Besides, the proportion of CD14^+^CD16^+^ monocytes and CD4^+^CD28^−^ T cells increases [23, 24]. Both subtypes exhibit notable proinflammation ability and are related to CVD in patients with ESRD [25, 26]. Moreover, the anti-bactericidal activity of neutrophils decreased, leading to increased susceptibility to infection. For instance, the ability of neutrophils to generate extracellular NET formation (NETosis) is severely impaired, favoring the invasion of pathogens [27]. In addition to causing complications, immune dysfunction also contributes to poor vaccine response in patients with ESRD [28]. It may be related to the decreased stimulation ability of dendritic cells (DCs) and impaired antigen-specific T cell differentiation [29].

## Effects of gut-derived metabolites on CKD

Uremic toxins can be divided into three categories: small water-soluble, protein-bound and middle molecules. Among them, several metabolites, such as IS, pCS and IAA, are in the limelight. All three are PBUTs. Unfortunately, they can hardly be removed by dialysis, because the molecule size of PBUTs exceeds the upper limit of dialysis membranes [30]. Moreover, HD cannot replace tubular secretion, which is the main mechanism of PBUTs excretion. Similar to HD, PD cannot compensate for the reduced renal function, and the restriction coefficient of its membrane for macromolecules increases over time [31]. PD even has a lower clearance of PBUTs than HD [32]. Besides, studies showed that hemodiafiltration (HDF) has no notable effects on removing PBUTs in the long term, even though its efficacy is higher than HD [33, 34]. Therefore, PBUTs accumulate in patients with CKD as the kidney function decreases, especially in advanced CKD stages.

Moreover, aside from toxins, gut microbiota can also produce metabolites with renoprotective properties such as SCFAs. The impacts of PBUTs and renoprotective metabolites are outlined in Table 1.

**Table 1:** Effects of gut-derived metabolites on kidney diseases

Protein-bound Uremic toxins	Effects	Refs.
Indoxyl sulfate	Increase of IL-1β and TNF-α in human monocytes	[37]
	Induction of TNF-α in macrophages	[39]
	Elicit adhesion and extravasation of leukocytes in mice	[41]
Indole-3-acetic acid	Damage of DNA and ultrastructural in neutrophils	[43, 44]
	Resulting in death of neutrophils	
p-Cresol sulfate	Increase of the percentage of free radical-producing leucocytes	[47]
	Inhibition of the production of IFN-γ in mouse T cells	[49]
	Inhibition of IL-12 in macrophages	[50]
	Suppression of the proliferation of CD43^+^ B-cell progenitors	[51]

Reno-protective metabolites	Effects	Refs.
Butyrate	Decrease of urine BUN and Scr levels; attenuation of renal pathology; reduction of macrophage infiltration and kidney inflammation	[60]
	Improvement of intestinal barrier and insulin sensitivity	[61]
Acetate	Retard of inflammation by inhibiting histone acetylation (HDAC) activity of T cells	[62]
Propionate	Alleviation of the increased Scr and BUN via the fatty acid receptor 2 (FFA2) or FFA3 pathway	[63]
	Inhibition of inflammation in HD patients	[64]

### Effects of PBUTs on immune cells

These accumulated toxins have multiple biological effects, of which the harmful effects on immune cells have gained more interest.

#### Indoxyl sulfate 

Indoxyl sulfate (IS) is metabolized from tryptophan. Tryptophan first is metabolized into indole by gut bacteria such as *Escherichia coli*, *Proteus vulgaris*, *Paracolobactrum coliforme*, *Achromobacter liquefaciens*, and *Micrococcus aerogenes* and then is hydroxylated and sulfated to circulating IS in liver [35].

The effects of IS on immune cells have been shown to contribute to kidney and vascular damage in patients with CKD. The tryptophan derivates are the ligands of aryl hydrocarbon receptor (AhR), which is expressed on kinds of immune cells and provides immune function [36]. Through the AhR pathway, IS induces human monocytes to produce interleukin (IL)-1β and tumor necrosis factor-α (TNF-α). IL-1β is involved in the onset and progression of tubulointerstitial fibrosis [37]. The increasing level of TNF-α can elicit the production of CX3CL1 in vascular endothelial cells, which will recruit CD4^+^CD28^−^ T cells that express CX3CR1. The activated CD4^+^CD28^−^ T cells cause endothelial cells to undergo apoptosis [38]. In macrophages, IS induces TNF-α production involving crosstalk among the AhR, NF-kB, and the suppressor of cytokine signaling (SOCS)2 [39]. In addition, IL-6, one of the pro-inflammatory factors, is proven to be notably related to the free IS level [40]. IS also induces adhesion and extravasation of leukocytes in a murine experiment, causing vascular impairment [41].

#### Indole-3-acetic acid 

Indole-3-acetic acid (IAA) also originates from tryptophan and belongs to the family of indole, which is metabolized by bacterial tryptophan monooxygenases and indole-3-acetamide hydrolases [42]. As a tryptophan derivate, IAA can also activate AhR. In addition, IAA has multiple effects on neutrophils, which may contribute to the development of infection. The production of ${\text{O}}_{2}^{-}$ and H_2_O_2_ activated by IAA leads to death and significant ultrastructural changes in cultured neutrophils [43]. IAA damages the DNA of neutrophils in a dose-dependent manner, resulting in the death of neutrophils [44]. Similar to IS, IAA can impair the vascular. In cultured human endothelial cells, IAA upregulates the expression and activity of COX-2 by activating an AhR/p38 MAPK/NF-κB signaling pathway and increases ROS production in endothelial cells, which induces endothelial inflammation and oxidative stress [45]. A study has demonstrated that serum IAA has predictive value for mortality and cardiovascular events in CKD [45].

#### p-Cresol sulfate 

p-Cresol is the product of tyrosine, most of which is sulfated to p-Cresol sulfate (pCS) in colon [46].

pCS exhibits both pro-inflammatory and immunosuppressive effects at different stages of CKD, which involves the progression of CKD and its complications. Schepers et al*.* showed the pro-inflammatory effect of pCS for the first time, which functions by increasing the percentage of free radical-producing leucocytes [47]. Similarly, Azevedo et al*.* described the elevated ability of NO production and phagocytosis in macrophages in the early stage of CKD. However, in advanced stages, the high concentration of pCS could cause immunosuppression [48]. Studies showed that pCS could inhibit the production of IFN-γ by decreasing Th1 cells and the production of IL-12 in macrophages, thus suppressing immune responses [49, 50]. In adenine-induced renal dysfunction mice, pCS could suppress the phosphorylation of STAT5, which is a vital process in the proliferation and survival of CD43^+^ B-cell progenitors. Therefore, pCS inhibited the proliferation of CD43^+^ B-cell progenitors [51]. The resulting reduction of B cells is associated with a decreased response to T-cell-independent vaccines in patients with ESRD [52]. The immunosuppression mechanisms may contribute to infection including septicemia, which is one of the leading causes of death in ESRD patients.

In addition to the reduced clearance, increased protein fermentation of gut flora also contributes to the elevated level of PBUTs. In CKD patients, the colon transit times are prolonged [53]. Moreover, the protein digestion of the upper gut is impaired, leading to the protein increase in colon [53]. A recent study linked gut flora in patients with CKD to uremic toxins levels. In this study, the abundance of *Escherichia_Shigella* is positively correlated with serum levels of IS; *Alistipes* is positively associated with total levels of IS and pCS [54].

### Effects of PBUTs on CKD progression

PBUTs are involved in fibrosis and inflammation in CKD. A recent study revealed that IS induces epithelial-to-mesenchymal transition (EMT) in human proximal tubular cells (HK-2), providing a potential mechanistic link to renal fibrosis [55]. Poveda et al. have reported that pCS causes cell death in HK-2 cells. The findings also showed that pCS increases expression of the TWEAK receptor Fn14 and cooperates with inflammatory cytokine TWEAK, collectively contributing to kidney damage [56]. Watanabe et al*.* explored the effects of pCS on renal tubular cells. They found that pCS led to the elevation of Nox4 and p22^phox^ expression, resulting in the increase of ROS production through Nox4 and p22^phox^ NADPH oxidase. Thereafter, ROS mediates the increase of pro-inflammatory and pro-fibrotic factors, ultimately contributing to damage in tubular cells [11]. PBUTs have been found to elicit comparable deleterious effects on cellular function. IS and pCS have been shown to inhibit the expression of Klotho in renal tubules of mice by DNA methylation, contributing to renal tubule senescence [57]. Moreover, both IS and pCS can elicit an increase in Tgfb1 expression in proximal renal tubular cells, which is related to immunomodulation and fibrosis [58]. The activation of RAAS is widely acknowledged as a critical mechanism driving the progression of CKD [16]. Both IS and pCS have been demonstrated to increase levels of renin, angiotensinogen and the expression of AT1 receptor, contributing to kidney injury [10]. Furthermore, it has been observed that uremic toxins can also affect metabolism in human renal proximal tubule cells. A study has revealed the involvement of both IAA and IS in reducing the activity of the mitochondrial electron transport chain, leading to decreased NAD + levels and subsequent attenuation of UDP-glucuronosyltransferases (UGT)-mediated metabolism [59].

### Reno-protective metabolites

In healthy individuals, gut microbiota ferments carbohydrates into SCFAs e.g., acetate, propionate and butyrate. Unfortunately, these microbiota-producing SCFAs are decreased in CKD. Notably, SCFAs have been attributed with a protective role in CKD, exerting anti-inflammatory effects, enhancement of gut barrier integrity, and amelioration of insulin sensitivity. Li et al*.* found that butyrate improves renal fibrosis, renal lesion and tubular injury through the GPR43 pathway. Moreover, it inhibits lipopolysaccharide-induced overexpression of proinflammatory *Il1b* and *Il6* in both kidney tubular epithelium cell and renal podocyte. The researchers observed reduced infiltration of macrophage in the kidney upon butyrate treatment [60]. In addition, Gonzalez et al*.* demonstrated the improvement of 5ʹ adenosine monophosphate-activated protein kinase (AMPK) phosphorylation by butyrate, resulting in enhanced gut barrier and reduced leakage of gut-derived uremic toxins. Butyrate mediates the secretion of glucagon-like peptide-1 (GPL-1), ameliorating insulin sensitivity [61]. Furthermore, acetate mitigates the inflammation in CKD by inhibiting the histone acetylation (HDAC) activity of T cells, correcting the oxidant-antioxidant imbalance in T cell [62]. As for propionate, researchers found that it alleviates the increased serum Cr (Scr) and BUN as well as inflammation via the GPR43 or GPR41 pathway [63, 64].

## Roles of the microbiome in CKD

### The impacts of the gut microbiome on CKD

#### IgA nephropathy

IgA nephropathy (IgAN) is the most prevalent disease among primary glomerulonephritis with 25 cases per 100,000 persons worldwide [65]. The pathogenesis of IgAN has been described as a multi-hit mechanism, the most important of which is the synthesis of galactose-deficient IgA1 (Gd-IgA1) [66]. Evidence has emerged suggesting the involvement of the gut microbiome in this pathogenetic process. Several studies have shown a reduction in cell densities and diversity of intestinal microbiota in IgAN patients when compared to healthy individuals [67, 68]. Additionally, research has indicated that *Sutterellaceae* and *Enterobacteriaceae* increase, both of which are LPS-producing bacteria [68]. In addition to inducing systemic inflammation, LPS significantly inhibits the expression of core I β3-Gal-T-specific molecular chaperone (Cosmc) mRNA and leads to methylation of the Cosmc by activating toll-like receptor 4 (TLR4) [69]. Cosmc is a critical molecular chaperone of IgA1 glycosylation [70]. Therefore, the decrease of Cosmc can cause the increase of circulating Gd-IgA1. The accumulated Gd-IgA1 can stimulate B cells to produce IgG, resulting in the sequential deposition of IgG-Gd-IgA1 immune complexes in kidney [71]. The complexes induce mesangial cell proliferation and the production of extracellular matrix components [66]. Moreover, in BAFF-overexpressing transgenic mice (BAFF-Tg mice), the presence of commensal microbiota is indispensable for IgA production and collaborates with high levels of BAFF to participate in the pathogenesis of IgAN [72]. Besides, the uremic toxins produced by imbalanced gut flora induce the release of pro-inflammatory cytokines and cause subsequent systemic inflammation, further contributing to the progression of IgAN [73].

Furthermore, a recent investigation has elucidated the correlation between intestinal microbiota and clinical manifestation of IgAN, shown by increased *Escherichia-Shigella* and reduced *Bifidobacterium* in patients with severe haematuria and proteinuria [74]. In another study, antibiotic administration leads to decreased formation of glomerular deposition and prevents proteinuria in a humanized mouse model of IgAN [75]. Therefore, targeting the gut microbiome may be a promising strategy to prevent the initiation and progression of IgAN.

#### Diabetic nephropathy

Approximately 40% of patients with diabetes that is poorly controlled develop diabetic nephropathy (DN), which has been the leading cause of CKD over the past years [76, 77]. DN is characterized by decreased glomerular filtration rate and increased albuminuria and seriously affects patients’ quality of life.

Current views hold that the activated RAS plays a pivotal role in renal dysfunction of DN [78]. The release of renin and Ang II can cause extracellular matrix accumulation and injury of renal cells [79]. The important metabolites of gut microbiota, SCFAs, are reported to participate in RAS regulation [80]. Acetate can activate olfactory receptor 78 (Olfr78) that is expressed on renal juxtaglomerular afferent arteriole, leading to the release of renin and subsequent increase in blood pressure [81]. In addition, acetate activates G protein-coupled receptor 43 (GPR43) to cause a disorder of cholesterol homeostasis, contributing to the lipid accumulation in the tubulointerstitium of DN patients [82]. The activation of GPR43 also mediates insulin resistance and thereby causes impairment in podocyte [83]. Podocyte injury is one of the vital mechanisms of albuminuria [84]. Moreover, insulin resistance (IR) also contributes to the progression of DN. IR leads to high insulin levels, which activates the production of plasminogen activator inhibitor 1 (PAI-1), thereby causing mesangial matrix expansion and fibrosis in kidney [85]. Administration of *Bifidobacteria* and *Lactobacillus* is observed to improve insulin resistance in type 2 diabetes mellitus (T2DM) [86].

Besides, hyperglycemia can impair the gut barrier, resulting in the increased translocation of gut microbiota and its products [87]. The increases of these metabolites in circulation exert negative effects on patients. For example, the upregulation of pro-inflammatory cytokines and inflammation can be caused by elevated LPS levels [88]. Moreover, LPS is associated with the occurrence of IR [88]. A recent study observed the translocation of gut microbiota and demonstrated its damage to kidney in mitochondrial antiviral signaling protein (MAVS) knockout diabetic mice [89]. MAVS is important in protecting the gut barrier [90]. Furthermore, phenyl sulfate (PS) is one of the gut microbiota-derived PBUTs, which increases in circulation of patients with DN [91]. Koichi et al*.* demonstrated that PS exerts multiple negative effects, including podocyte injury, mitochondrial dysfunction of the podocyte, and fibrosis in kidney [91]. This study also suggests that PS levels may serve as a predictive biomarker for the progression of albuminuria for incipient patients with DN [91]. In addition to kidney injury, PBUTs also contribute to the complication of DN. IS induces the proliferation of vascular smooth muscle cells, indicating its role in the development of CVD in patients with DN [92].

#### Acute kidney injury to CKD transition

Acute kidney injury (AKI) is defined as the sudden declination of renal function within a few hours or days. The maladaptive repair in AKI can predispose individuals to progress toward CKD, particularly in the context of kidney aging. Previous studies suggest that gut dysbiosis in AKI accelerates the transition from AKI to CKD. In vitro experiment, Chen et al*.* demonstrated that IS is associated with hypoxia–reperfusion (H/R) induced G2/M cell cycle arrest, EMT and endoplasmic reticulum stress induction, which may lead to the maladaptive repair of AKI [93]. The cells that are arrested in the G2/M phase may secret pro-inflammatory factors, leading to microvascular rarefaction and collagen disposition in kidney [94]. Moreover, AKI is characterized by severe proximal tubule injury, including the downregulation of organic anion transporter (OAT)-1 and OAT-3, subsequently causing the decreased excretion of uremic toxins such as IS. In addition, the impaired gut barrier caused by both AKI and dysbiosis can indirectly expedite the transition from AKI to CKD. Butyrate is able to activate hypoxia-inducible factor (HIF) -1α in intestinal epithelial cells (IECs). HIF-1α promotes mucin expression and secretion, therefore maintaining the stability of gut barrier [95]. However, the SCFAs are reduced due to gut dysbiosis in CKD, compromising gut barrier and causing the leakage of gut-derived uremic toxins. Yang et al*.* demonstrated that AKI-induced dysbiosis can contribute to the progression of AKI. The results showed that dysbiosis is associated with increased colonocyte apoptosis, inflammation, and altered tight junction proteins, which may lead to the translocation of endotoxins and further potentiate the inflammation in AKI [96, 97].

#### Hemodialysis/Peritoneal dialysis

ESRD patients require renal replacement treatment (RRT), such as hemodialysis (HD) and peritoneal dialysis (PD). HD and PD retard the progression of ESRD through clearance of the retention water and removal of some uremic toxins, consequently maintaining the balance of the internal environment.

Infection is an important complication, both in HD and PD. Studies suggest that the gut microbiota may be a source of infectious pathogens. For example, *Pseudomonas aeruginosa* and *Enterobacteriaceae* increase in PD patients [98, 99]. *P. aeruginosa* has been found to be associated with around 40% of catheter removals attributed to infections, while *Enterobacteriaceae* is responsible for 12.0% of peritonitis cases in PD patients [100, 101]. Moreover, the DNA of *P. aeruginosa* was found in blood from different vascular access of HD patients [102].

During dialysis, the intestinal barrier is compromised. Increased intraperitoneal pressure in PD contributes to intestinal hypoperfusion [103]. Similarly, HD is always accompanied by reduced hepato-splanchnic perfusion, which leads to intestinal ischemia [104]. Therefore, the subsequent migration of bacteria and its metabolites exerts negative effects on HD and PD patients. For instance, circulating IS and pCS are elevated in HD and PD patients. The harmful effects of IS and pCS on the human body have been reviewed elsewhere, such as endothelial damage, EMT and inflammation [105]. Based on these mechanisms, researchers find evidence that pCS is associated with the morbidity of CVD and all-cause mortality in HD patients [106]. Moreover, pCS and IS can predict the progression of CKD [107].

In addition, constipation is common in dialysis patients. The decrease of commensal bacteria can contribute to the occurrence of constipation. *Lactobacillus* and *Bifidobacterium* are associated with faster colonic transit [108]. However, they decreased in PD patients, which may confer negative effects on gut healthy [109]. The mechanisms of intestinal microbiome progress the disease is enormously complex and only partly understood. We need more research to unravel the mysteries.

### Microbiota in CKD

The gut microbiome is different in healthy individuals and patients with CKD. Research has revealed an elevated presence of both aerobic and anaerobic microorganisms in the duodenum and jejunum of CKD patients compared to healthy individuals [110, 111]. Moreover, many results demonstrate a notable reduction in the diversity of gut microbiota among CKD patients [5, 112]. Furthermore, a recent study conducted in China enrolled 489 fecal samples to find the alterations in the gut microbiota of patients with CKD compared to healthy individuals [113]. As CKD progressed, researchers observed significant increases in the abundance of genera *Thalassospira*, *Akkermansia*, and *Blautia*, while the genus *RF9_norank* exhibited a decrease. Particularly, *Akkermansia* is positively correlated with BUN and Scr, indicating its potential as a valuable diagnostic or therapeutic target in CKD. Moreover, the microbiota involved in the metabolism of ascorbate and aromatic amino acids is enriched in CKD, which aligns with the observed increase in uremic toxins in CKD patients [113].

In addition to the gut, the composition of microbiota in blood holds significant importance. Blood is traditionally supposed to be sterile, however, a recent study challenged this view, providing evidence supporting the existence of blood microbiome in healthy individuals [114]. Shah et al*.* compared the blood microbiome of patients with CKD and healthy controls. The results showed that the α diversity is decreased in the CKD group, similar to the change in the gut microbiota of CKD. Moreover, *Proteobacteria* is enriched in the CKD group. This bacteria phylum increased in blood and gut in many chronic diseases. Therefore, decreased diversity of blood microbiota and enriched *Proteobacteria* in the blood may be the factors contributing to CKD progression [115]. Merino-Ribas et al*.* explored the blood microbiome in PD patients with or without vascular calcification. They found an increase in *Devosia* in the blood of PD patients with vascular calcification. Vascular calcification is associated with high mortality risk in PD patients, suggesting that *Devosia* may be a biomarker or involved in the disease progression [116]. However, many questions remain to be addressed. The precise origin of blood microbiota has yet to be definitively determined. The leaky gut barrier and translocation of microbiota in oral and skin may be the sources [117]. Moreover, liver clearance and the immune system have important effects on the blood microbiome, emphasizing the necessity of incorporating these factors in future investigations [118].

The research on urine microbiome in patients with CKD is still not fully explored. A study enrolled 77 patients with non-dialysis-dependent CKD in order to understand the characteristic of urine microbiome in stage 3–5 CKD. The findings revealed a reduction in the diversity of the midstream urine microbiome concomitant with the decline in eGFR [119]. The possible mechanism may be the altered secretion of uromodulin, which favors the excretion of bacteria [119]. Since secondary urinary tract infection is common in patients with CKD, more attention should be paid to the urine microbiome.

### Gut-kidney-heart axis

Among the complications of CKD, CVD stands as a prominent cause of mortality in CKD patients. The pathogenesis of CVD in CKD is multifactorial, involving several mechanisms, including (1) volume overload; (2) overactivation of RAAS; (3) inflammation and oxidative stress [120]. The detailed interaction between the heart and kidney in CKD has been well reviewed elsewhere [121]. Additionally, the gut microbiome also precipitates the development of CVD, particularly by producing uremic toxins. Thereafter, accumulated gut-derived uremic toxins are transferred to circulation through the impaired gut barrier and compromise the cardiovascular system.

The gut-derived uremic toxins exhibit the capacity to provoke vascular damage and promote the development of atherosclerosis. Gut bacteria metabolize choline, choline-containing compounds, betaine, and l-carnitine into trimethylamine (TMA), which is oxidized by flavin-dependent monooxygenase isoforms 1 and 3 (FMO1 and FMO3) to form TMAO in liver [122]. Seldin et al*.* have elucidated the capacity of TMAO to upregulate the expression of inflammatory genes in endothelial and smooth muscle cells by activating the NF-κB signaling pathway. Moreover, TMAO enhances the endothelial adhesion of leukocytes and contributes to atherosclerosis [123]. In addition, both TMAO and LPS promote atherosclerosis by increasing the expression of osteopontin (OPN) and activating macrophages of aortic [124]. OPN is an independent traditional risk factor of CVD [125]. In addition to TMAO, both IS and IAA possess the ability to increase the expression of TF protein via the AhR pathway, which is associated with several cardiovascular diseases such as atherosclerosis [126]. As reported by Campillo et al*.*, IS and p-cresol can lead to monocytes across the extracellular matrix (ECM) by activating the integrin-linked kinase (ILK) /AKT signaling pathway. The activation of ILK/AKT signaling results in the formation of podosome, which is involved in ECM degradation. The increased degradation of the matrix favors the migration of monocytes and the interaction between migrated monocytes and vascular may cause injury [127].

The vicious cycle among the gut, kidney and heart is intricate, and only a portion of mechanisms is currently elucidated. In addition to metabolites, how gut microbiota itself affects the gut-kidney-heart axis is needed to explore. Nevertheless, targeting this vicious cycle to develop novel interventions holds tremendous potential for yielding significant benefits for patients.

## Advances in techniques and methodologies for gut microbiome in CKD research

In the 1970s, scientists detected gut microbiota by culture–based techniques [128]. However, most of the gut flora cannot be cultured in the experimental condition. In the past decades, sequencing technologies and data analysis pipelines develop rapidly and are applied widely, enabling us to put insight into the composition, and capability of the human intestinal microbiome [129]. The pros and cons of technologies have been reviewed elsewhere [130].

With the advance of tools, the understanding of gut microbiome in patients with CKD has been improved a lot. Numerous scholars have reported the composition of gut microbiota by analyzing 16S rRNA sequence data. Compared with healthy control, *Klebsiella* and *Enterobacteriaceae* increased, while *Blautia* and *Roseburia* decreased in patients with CKD [113]. Further, Wu et al*.* showed the differences in gut microbiota within different CKD stages. *Escherichia_Shigella* is significantly enriched in advanced CKD, while *Dialiste*, *Lachnospiraceae_ND3007_group*, *Pseudobutyrivibrio*, *Roseburia*, *Ruminiclostridium* spp. decrease with CKD progression [54]. In addition, the comparison between pre-dialysis and dialysis patients is also worthy of attention. A recent study showed that dialysis increases some commensal bacteria and elicits some pathogenic bacteria at the same time compared with non-dialysis ESRD patients [131]. The shotgun approach is sequencing all microbial genomes in the samples. It can provide data with higher resolution than 16S rRNA sequencing and help understand the metabolic activity of microbiota [132]. Recently, Wang et al*.* explored the taxonomic and function of the microbiome in patients with ESRD utilizing shotgun metagenome sequencing. They found that the amino acid biosynthesis is decreased and amino acid degradation is increased. Moreover, the enzyme involved in aromatic amino acid degradation and secondary bile acid biosynthesis is enriched. This evidence may suggest the source of accumulated uremic toxins in ESRD [133].

Besides, multi-omics analyses combined data from multiple biological sources such as genomics, metagenomics, transcriptomics, proteomics, and metabolomics promote a mechanistic understanding of gut-kidney axis on CKD progression. Opdebeeck et al*.* found that IS and pCS can induce calcification in aorta and peripheral arteries of rats. The proteomic and bioinformatic analysis identified the proteins in these vessels suggesting that inflammation, coagulation and glucometabolism pathways are correlated with the IS- and pCS-induced calcification [134]. In the metabolomics study, Wu et al*.* also found that caproic acid is highly positively correlated with eGFR, indicating its role in alleviating the severity of CKD [112]. Using a proteomics approach, Karaduta et al*.* found that CKD mice exhibited a similar pattern of gut proteins as shown in healthy mice after receiving resistant starch. They further verified the protective role of resistant starch in renal injury [135]. Lobel et al*.* also applied proteomics to study the relationship between diet, gut and kidney. They identified that TnaA is a highly S-sulfhydrated protein, which is able to catalyze tryptophan to indole. They demonstrated that high sulfated amino acid dietary can inhibit the activity of TnaA by posttranslational modification, reducing the production of the IS precursor and consequently alleviating the progression of CKD [136]. By integrating these different omics data, researchers can attain profound insights into the intricate molecular mechanisms involved in microbiome-host crosstalk.

In addition to exploring the relationship between gut microbiome and CKD, omics contribute to finding the causes and novel diagnosis markers of various kidney diseases. Sethi et al*.* analyzed the proteomic profile of 12 cases of C3 glomerulonephritis (C3GN). The results showed that alternative pathway (AP) dysregulation can lead to C3GN by causing glomerular accumulation of AP and TCC proteins [137]. Dasari et al*.* detected proteins in glomerular biopsy specimens of patients by liquid chromatography and tandem mass spectroscopy (LC–MS/MS). They found that DnaJ homolog subfamily B member 9 (DNAJB9) is a specific protein in fibrillary glomerulonephritis, which indicates the role of DNAJB9 in pathogenesis and its potential to be a biomarker [138]. Moreover, metabolomics is used to explore the underlying metabolic pathway in CKD progression. Ma et al*.* revealed that the overexpression of *APOL1* gene, previously demonstrated to be associated with CKD progression, can lead to alterations in the tricarboxylic acid cycle, increased fatty acid oxidation, and compromised redox homeostasis [139].

## Potential therapies

Given the close interplay between the gut microbiome and CKD, some therapies target bacteria and its metabolites, which may alter the gut milieu and ameliorate the progression of CKD and its complications.

### Probiotics

Probiotics are living microorganisms. It has been accepted that probiotics confer health benefits on gut environment. The underlying mechanisms for the beneficial effects of probiotics on gut health are multifaceted and include the following: (1) Immunomodulation, where specific bacterial strains can exert anti-inflammatory effects by inhibiting NF-κB signaling [140]; (2) Enhancement of the intestinal barrier, as probiotic treatment has been shown to inhibit changes in tight junctions (TJs), which are vital components of the gut barrier [141]; and (3) Resistance to pathogens, as *Bacillus subtilis LF11* has been demonstrated to protect epithelial cells by reducing the attachment and invasion of *Salmonella* [142]. Some probiotics secret bacteriocins to play an antimicrobial role [143].

There are studies showing the beneficial effects of probiotics on CKD. Simenhoff et al*.* treated 8 HD patients with oral *Lactobacillus acidophilus* and the results showed reduced serum dimethylamine (DMA) which damages the organ vascular [17, 110]. Moreover, oral administration of *Bifidobacterium longum* in capsule reduced serum IS levels [144]. Recently, Zhu et al*.* investigated the therapeutic effects of the probiotic *Lactobacillus casei Zhang* in both mouse models and patients with CKD. The findings revealed that the administration of *L. casei Zhang* improved the inflammatory response of local macrophages and tubular epithelia cells by increasing the level of serum SCFAs [145]. Further, the study observed a slower decrease in renal function in patients with CKD stages 3–5 after taking *L. casei Zhang* [145]. However, due to the extensive variation in diet, disease condition and age of individuals, the therapeutic precision of the probiotics needs to be fully considered.

### Prebiotics

Prebiotics are non-digestible food ingredients that improve human health by stimulating the growth of commensal bacteria, such as *Lactobacillus* and *Bifidobacterium* [146]. Common prebiotics include resistant starch, inulin, and oligosaccharides such as fructo-oligosaccharides (FOS) and galacto-oligosaccharides (GOS) [147]. The prebiotics can be metabolized by gut microbiota to produce SCFAs, which in turn can exert beneficial effects. SCFAs play a crucial role in providing an energy source of gut microbiota, regulating the luminal pH and inhibiting the growth of pathogens [148]. Furthermore, GOS protects disrupted gut epithelial and accelerates the TJ reassembly [149]. Oligofructose inulin treatment for four weeks reduced the serum level of pCS in HD patients, with the effect lasting for at least four weeks [150]. Moreover, the association between the ratio of total protein to total fiber and the levels of both IS and pCS is found to be significant, and incorporating this index into the eGFR regression model can improve its predictive power for the levels of uremic toxins [151].

In addition to non-digestible food ingredients, oral sorbents are used to reduce toxins levels. AST-120 is a sorbent with porous carbon particles. Several reports have demonstrated its beneficial effects on ameliorating CKD progression. A study showed that AST-120 reduces serum IS levels and ameliorates oxidative stress in cardiac tissue in CKD rats [152]. Recently, Huang et al*.* demonstrated that IS could inhibit mitophagy and subsequently cause damage to the intestinal barrier [153]. Mitophagy plays a protective role in healthy cells [154]. The study further revealed that AST-120 can provide protection against mitophagic impairment and intestinal barrier injury in CKD mice models [153]. Furthermore, the efficacy of AST-120 has also been demonstrated in human studies. A study by Nakamura et al*.* enrolled fifty patients with chronic renal failure, and the effects of AST-120 therapy were evaluated [155]. The results revealed that AST-120 therapy significantly reduced the levels of IL-6 and proteinuria, and inhibited the increase of serum creatinine levels [155]. However, it was observed that the therapy was not effective in preventing the decrease of GFR [155]. More clinical studies have been reviewed elsewhere [156]. Because of the challenges associated with accurately determining the sample size and duration of the study necessary, the evaluation of the effects of AST-120 on the renal hard endpoint is insufficient in many studies. Therefore, more well-designed studies need to be performed.

### Synbiotics

Multiple combinations of probiotics and prebiotics have been applied in clinical practices, including OAT fiber/*L. plantarum*, and FOS/*L. sporogens* [157]. Synbiotics have the collaborative effects of the prebiotics and probiotics, which has higher efficacy. Probiotics are capable of releasing bacteriocins that inhibit bacteria involved in p-cresol production, while prebiotics can promote the growth of commensal bacteria [158]. A study has reported the efficacy of Probinul-neutro^®^ in reducing serum p-cresol levels in patients with CKD when administered thrice daily for four weeks [159]. Another single-center, double-blind, placebo-controlled, randomized crossover trial study has found that synbiotics significantly reduce serum pCS but not IS in patients with CKD [160]. Moreover, synbiotics can increase *Bifidobacterium* and decrease *Ruminococcaceae* in their stool microbial community [160]. Recently, synbiotics have been shown to improve dysbiosis, decrease the amount of fecal indole and ameliorate CKD progression in CKD rat models [161].

### Dietary manipulation

A current well-accepted hypothesis is that high-protein diets may be harmful to kidney [162]. The underlying mechanisms include causing hyperfiltration in kidney and upregulating the renal expression of TNF-α and IL-6 [163, 164]. In the Singapore Chinese Health Study, the researcher found that ESRD risk increases with an increased intake of red meat [165]. Moreover, most foods with high protein contain phosphate, which will disturb the serum phosphate level in the early stages of CKD [166]. On the contrary, the restriction of dietary protein can reduce sclerosis in kidney [167]. Therefore, many experts recommend a plant-dominant low-protein diet for patients with CKD [168]. In a meta-analysis, researchers found that CKD patients had lower mortality when adopting a healthy diet rich in fruit and vegetables, fish, legumes, cereals, whole grains, and fiber, and less in red meat and products containing sodium and refined sugars [152]. Furthermore, high-fiber diets contribute to the growth of beneficial bacteria such as *Bacteroidetes* that are capable of producing SCFAs [153]. These SCFAs can improve gut dysbiosis. Given the powerful role of diet–microbiota–host crosstalk, dietary manipulation could be an effective therapeutic tool [154].

### Genetic engineering

In 2015, Sonnenburg proposed the concept of ‘‘smart’’ bacteria that can secrete anti-inflammatory molecules [169]. In recent years, genetic engineering technology has been also applied to reduce toxins and ameliorate disease. Devlin et al*.* discovered a family of tryptophanases widely distributed in the gut commensal *Bacteroides* [170]. Through the deletion of this gene, they were able to eliminate indole production in vivo [170]. Moreover, researchers have successfully designed microencapsulated genetically engineered live *E.coli* DH5 cells [171]. They demonstrated the effective reduction of urea levels in uremic rats upon oral administration of the products [171]. Besides, Steidler et al*.* designed *Lactococcus lactis* to secret IL-10 in the colon [172]. When this engineered *Lactococcus lactis* was orally administered, it was found to have a beneficial effect on reducing inflammation in colitis mouse models [172]. Recently, Ting et al*.* showed an innovative method to deplete a specific bacterium from gut flora. They designed *E. cloacae* strains expressing nanobodies, called programmed inhibitor cells (PICs) [173]. PICs recognize target bacteria via surface nanobodies and eradicate it from gut microbiota [173]. Even though microbiota engineering is far from clinical application, it may be a promising way to achieve precision medicine.

### Fecal microbiota transplantation

Fecal microbiota transplantation (FMT) involves the transfer of gut microbiota from a healthy donor to a patient by infusing donor feces into the patient. It aims to restore the disturbed gut microbiota in patients. Therapeutic effects of FMT have been first demonstrated in the treatment of recurrent *Clostridium difficile* infection (CDI) [174]. The US Food and Drug Administration (FDA) permits the administration of FMT to CDI that does not respond to standard therapies [175]. Subsequently, there are increasing reports of the efficacy of FMT on other diseases [176]. A study found that FMT can decrease albuminuria and modulate renal phenotype in antibiotic-treated humanized IgAN mice [177]. In addition to effectiveness, a study has investigated the occurrence of adverse events (AEs) of FMT. A systematic analysis of cases published between 1983 and 2015 suggested an overall incidence of AEs was 28.5% (310/1089) [178]. Most of the AEs were mild, including abdominal discomfort, diarrhea, transient fever, nausea, vomiting and constipation [178]. The findings indicate that FMT is generally safe. However, rare but severe AEs have been reported, such as death, severe infections, and relapse of inflammatory bowel diseases (IBD) [178]. The risk of infections is associated with the inability of screening latent infection of donors [178]. Therefore, FDA has issued a warning on the risk of FMT [179]. More rigorous studies are required to assess the short- and long-term risks and efficacy of FMT.

## Challenges and perspectives

As a result of technological advancements, the intricacies of the gut microbiome have been partly elucidated, revealing the involvement of gut microbiota and its metabolites in the progression of CKD. In particular, PBUTs have been identified as contributors to immune dysfunction with adverse effects on CKD and its complications. Thus, novel interventions that aim to mitigate or even eliminate these detrimental effects should be developed. As noted, genetic engineering is a promising technology. Targeting enzymes that involve in PBUTs production by utilizing genetic engineering may be a feasible avenue. Integrating this approach with the assessment of efficacy in ameliorating immune dysfunction and CKD progression may help with the design of therapeutic strategies.

Current treatments aim to reduce the levels of PBUTs and regulate dysbiosis through dietary restriction and synbiotics. However, these therapies are limited by inter-individual variabilities in diet, lifestyle, age, and medication. Therefore, personalized therapeutic strategies based on a patient’s status are needed. Long-term and continuous follow-up is also required to yield clinically meaningful results. Despite these challenges, the gut microbiome in patients with CKD represents an exciting area of research. The microbiome-based treatments show promise in improving clinical practice and outcomes of patients with CKD.

## Data Availability

Not applicable.
